# Chromatin proteins and RNA are associated with DNA during all phases of mitosis

**DOI:** 10.1038/celldisc.2016.38

**Published:** 2016-10-25

**Authors:** Kathryn L Black, Svetlana Petruk, Tyler K Fenstermaker, Jacob W Hodgson, Jeffrey L Caplan, Hugh W Brock, Alexander Mazo

**Affiliations:** 1 Department of Biochemistry and Molecular Biology and Kimmel Cancer Center, Thomas Jefferson University, Philadelphia, PA, USA; 2 Department of Zoology, University of British Columbia, Vancouver, BC, Canada; 3 Delaware Biotechnology Institute Bio-Imaging Center, University of Delaware, Newark, DE, USA

**Keywords:** chromatin, epigenetics, mitosis, PcG, TrxG

## Abstract

Mitosis brings about major changes to chromosome and nuclear structure. We used recently developed proximity ligation assay-based techniques to investigate the association with DNA of chromatin-associated proteins and RNAs in *Drosophila* embryos during mitosis. All groups of tested proteins, histone-modifying and chromatin-remodeling proteins and methylated histones remained in close proximity to DNA during all phases of mitosis. We also found that RNA transcripts are associated with DNA during all stages of mitosis. Reduction of H3K27me3 levels or elimination of RNAs had no effect on the association of the components of PcG and TrxG complexes to DNA. Using a combination of proximity ligation assay-based techniques and super-resolution microscopy, we found that the number of protein–DNA and RNA–DNA foci undergoes significant reduction during mitosis, suggesting that mitosis may be accompanied by structural re-arrangement or compaction of specific chromatin domains.

## Introduction

DNA replication [1, 2] and mitosis [3–5] are the two phases of the cell cycle during which epigenetic marks must be inherited. Although mitosis is characterized by increased chromosome condensation, the accompanying molecular mechanisms are poorly understood at the chromatin level. A recent study assaying genome-wide DNase I accessibility (Hsiung *et al.* [6] and references therein) suggests that changes during mitosis are gene-specific and favor promoter proximal regions and that chromatin compacts 2–3-fold during mitosis. Mitosis is accompanied by genome-wide cessation of transcription [7] at early mitotic stages [8] and is associated with global phosphorylation of transcription factors and histone H3 (H3Thr3 and H3Ser10) [9, 10], as well as deacetylation of histones [11].

Epigenetic bookmarking during mitosis is thought to include modified histones, histone modifiers, nucleosome remodeling and transcriptional machineries, transcription factors and non-coding (nc) RNAs [12–15]. Some of these proteins dissociate during all or some stages of mitosis, while others remain on mitotic chromosomes. Until recently, the fate of transcripts during mitosis was unknown. However, we showed that in human lymphoblast cells RNAs are stable through all stages of mitosis [16].

The current methods for studying association of proteins with chromosomes in mitosis are limited to two major approaches: immunofluorescence (IF) detection of colocalization of proteins with DNA and chromatin immunoprecipitation (ChIP) assays [17]. Although IF can detect proteins at all mitotic stages, ChIP studies are limited to early stages, as they use cells synchronized at early mitotic stages with inhibitors of microtubule formation. The divergence in the IF-based experimental results for chromosome association during mitosis is especially marked for the PcG and TrxG proteins that are required for maintenance of gene repression and activation, respectively, during development [18]. They regulate their target genes by binding to promoter proximal regions or to promoter distal sites termed PcG response elements and TrxG response elements, respectively [19]. They function at these sites as protein complexes of chromatin-remodeling factors, histone-modifying enzymes or polynucleosome compaction factors [20] in modulating chromatin structure.

Paradoxically, most PcG proteins dissociate at metaphase, during which maximal chromosome condensation occurs. IF and ChIP assays using *Drosophila* cell lines suggested retention of PcG proteins PSC, PC and dRING/SCE on mitotic chromosomes, although the levels of these proteins were lower compared with their association with interphase DNA [5]. Similarly, IF analysis of mammalian cells detected association of PcG proteins SUZ12 and EZH2 and histone H3K27me3 with mitotic chromosomes [21]. In contrast, Buchenau *et al.* [3] showed a loss of PC and reduced PSC by IF in *Drosophila* embryos. The re-association of PcG proteins with decondensed chromosomes at anaphase/telophase was suggested to result from re-assembly of PcG complexes [3].

During mitosis, some TrxG proteins remain associated with condensed chromosomes and may be retained for gene activation in telophase or upon exit from mitosis [13, 22]. Analysis of association of TrxG proteins with mitotic chromosomes is also controversial: while some IF studies detected MLL1 on mitotic chromosomes [13, 23, 24], Mishra *et al.* [25] showed that MLL1 is displaced during mitosis. Using ChIP assays, Blobel *et al.* [13] found that while MLL1 associates with mitotic DNA, other TrxG proteins, H3K4 histone methyltransferases (HMTs) MLL2, SETD1, ASH2L and H3K4 demethylase LSD1 are displaced from chromatin during mitosis. These conflicting reports on the detectable levels of TrxG and PcG proteins associated with condensed chromosomes at prophase and metaphase [3, 5, 26, 27] may arise from the technical limitations of the assays, cell synchronization methods or developmental stage of the examined tissues.

Here we used two recently developed novel techniques to determine the association of chromatin proteins [28] and RNAs [16] with DNA during mitotic stages. These assays are based on the detection of proteins or RNA on DNA by proximity ligation assays (PLA). Using these assays, we show that RNAs and multiple chromatin proteins, including PcG and TrxG histone-modifying enzymes, chromatin-remodeling factors and major forms of methylated histones remain associated with a limited number of foci on DNA during all phases of mitosis. Furthermore, we found that H3K27me3 and RNAs are not essential for association of PcG and TrxG proteins with DNA during any stage of mitosis.

## Results

### Methylated histones, histone-modifying and nucleosome-remodeling proteins remain associated with DNA during all stages of mitosis

Previously we developed a PLA-based Chromatin Association Assay (CAA) that detects close proximity of a protein to 5-ethynyl-2′-deoxyuridine (EdU)-labeled DNA *in vivo* [28]. Using CAA and other assays, we found that, in *Drosophila* embryos, major methylated histone forms H3K27me3 and H3K4me3 are replaced during replication with unmodified histone H3 [28]. Similarly, H3K4me1, H3K4me2, H3K9me3, H4R3me2, H3R17me2 and H3K27Ac were displaced during replication and were accumulated at nascent DNA with various delays after DNA replication [29]. These results suggested that modified histones are unlikely to carry epigenetic information through DNA replication.

Here, using CAA, we asked whether modified histones are associated with DNA during mitosis. PLA has a unimolar sensitivity (10^−40^ M, Olink) and CAA detects very close proximity (40 nm, Olink) of a protein to DNA. Therefore, compared with conventional IF, this method provides a direct and very sensitive approach to detect association of proteins with labeled DNA at all stages of mitosis. We used CAA in *Drosophila* embryos at nuclear divisions 14–16 to detect the association of proteins with DNA during mitosis. At this developmental stage, the cell cycles are very short (S phase is 50 min, G2 is from 30 to 150 min and M is about 10 min) and lack the G1 interphase. DNA was labeled with EdU for 30 min followed by a 2 h chase; this allowed EdU-labeled cells to enter into mitosis. EdU was subsequently conjugated to biotin, and antibodies to biotin and tested protein were then used in PLA to examine the proximity of a protein to labeled DNA as described previously [28, 29]. Following PLA, embryos were counterstained with antibody to histone H3 phosphorylated at serine 10 (H3S10-p, a specific marker for mitosis) to visualize mitotic stages and with antibody to biotin to detect EdU-labeled nuclei.

**Figure 1:**
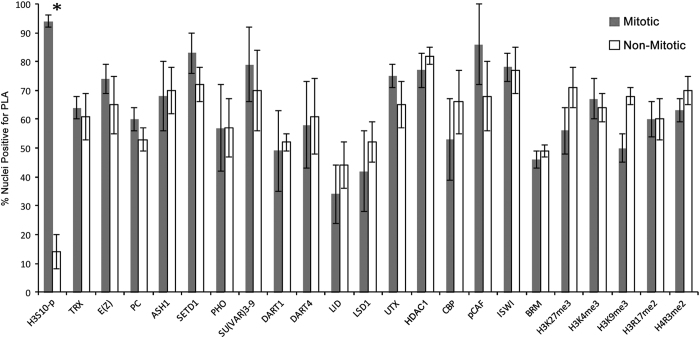
Comparison of association of chromosomal proteins and histones with DNA during interphase and mitosis. Statistical comparison of nuclei with positive proximity ligation assay (PLA) signals in interphase versus all stages of mitosis. All proteins/histones tested, except H3S10-p (*P<0.05 determined by two-tailed independent *t*-test), have similar association with DNA during all phases of mitosis. Note: For HP1 and OSA (presented in Figure 2c and d), we were unable to accurately quantify the percentages of PLA-positive nuclei in mitosis versus interphase nuclei because their antibodies are from the same species as antibody to the mitotic marker H3S10-p. Error bars represent s.d.

**Figure 2:**
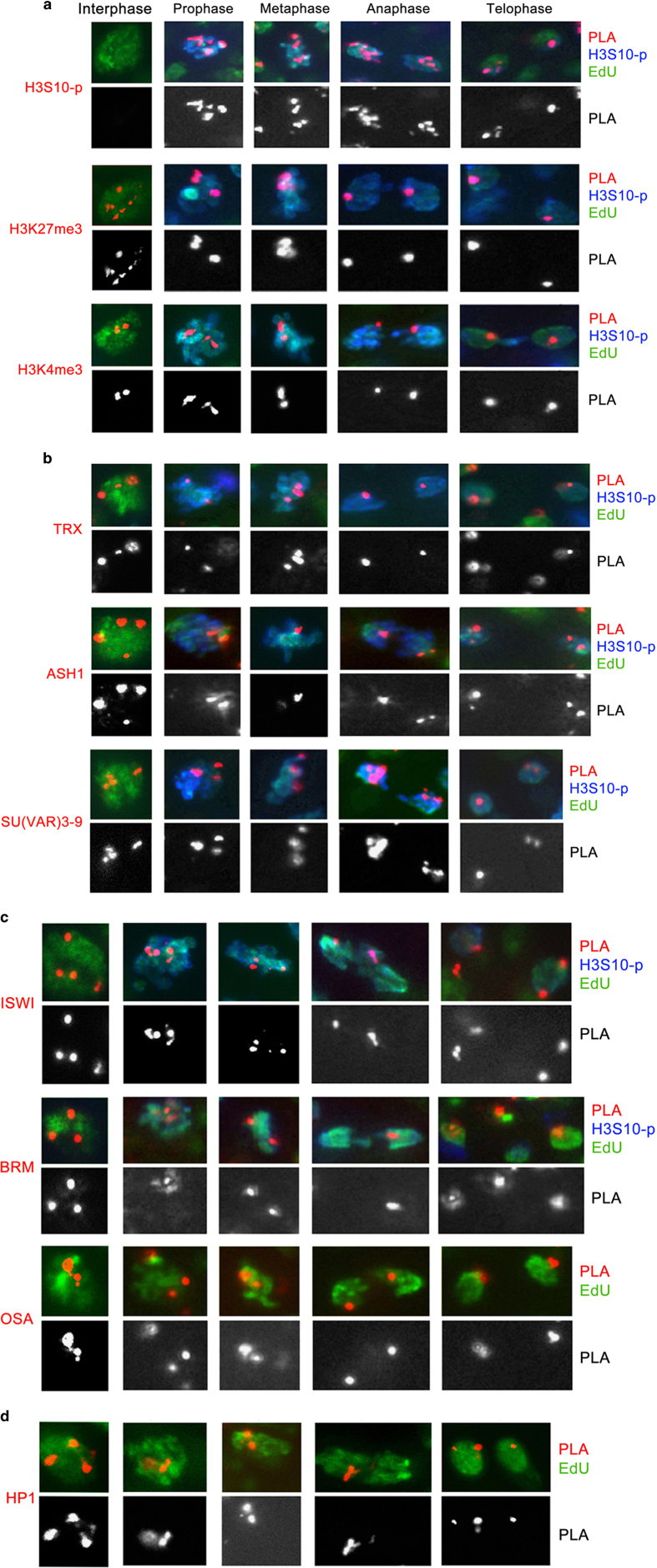
Modified histones, histone-modifying enzymes and histone-remodeling proteins are associated with DNA during mitosis. Proximity of the modified histones H3S10-p, H3K27me3, H3K4me3 (**a**), TRX, ASH1, SU(VAR)3–9 (**b**), ISWI, OSA, BRM (**c**) and HP1 (**d**) to DNA during interphase and all phases of mitosis was tested by Chromatin Association Assay. Proximity ligation assay (PLA), red; H3S10-p, blue; 5-ethynyl-2′-deoxyuridine (EdU), green. H3S10-p is a marker for mitosis and serves as a positive control. Note: As antibodies against HP1 and OSA are from the same species (mouse) as antibody to H3S10-p, H3S10-p marker was not used in these experiments. PLA, red; H3S10-p, blue; EdU, green. PLA signals only are shown in the bottom black and white panels.

Since in these experiments the cell cycle is not synchronized, and DNA in each nucleus may be labeled with EdU for varying times and at different stages of S phase, some EdU-labeled nuclei may lack PLA signals. Therefore, to quantify potential changes in association of these proteins with DNA during mitosis, we compared the total number of EdU-labeled nuclei with the number of EdU-labeled nuclei positive for PLA signals on interphase and mitotic chromosomes. All tested modified histones and chromosomal proteins were detected with similar efficiency during interphase and mitosis (Figure 1). In control experiments, PLA signals between a mitotic marker H3S10-p and EdU-labeled DNA are detected at all stages of mitosis but were only occasionally detected in the interphase nuclei (Figures 1 and 2a and Table 1).

The quantification of the results of these experiments for the interphase and mitotic phases is presented in Table 1 and images for representative groups of proteins are shown in Figure 2a–d. Our results suggest that major methylated histones forms, H3K27me3, H3K4me3, H3K9me3, H4R3me2 and H3R17me2, which are associated with EdU-labeled DNA in interphase, remain associated with DNA at all four stages of mitosis. These results are in contrast to the absence of these histone forms using CAA during early stages of DNA replication [28, 29] and suggest that methylated histones may have an epigenetic role during mitosis.

With the exception of the H3K4 HMT ASH1 and H3K9 HMT SU(VAR)3–9, all other tested histone-modifying proteins were detected in close proximity to the replication complex and on short nascent DNA [28, 29]. Here we found that TrxG proteins, lysine HMTs TRX, ASH1, and SETD1; the PcG proteins PHO, PC and lysine HMT E(Z); and suppressor of variegation H3K9 HMT SU(VAR)3–9, as well as the arginine HMTs DART1 and DART4, remain associated with DNA during all stages of mitosis (Figures 1 and 2b and Table 1). We found that the lysine demethylases LID, LSD1 and UTX, which counteract the activities of lysine HMTs, are also stably associated with DNA in mitosis. Similarly, other groups of histone-modifying enzymes, histone acetyltransferases CBP and PCAF and histone deacetylase HDAC1, were also found to be stably associated with mitotic DNA (Figure 1 and Table 1).

TrxG proteins BRM, OSA and ISWI are components of six major chromatin-remodeling complexes. Our previous CAA-based data suggested that these proteins are displaced from DNA during replication and are recruited back from 10 min to 2 h after replication [29]. In contrast, we found that all these proteins are associated with mitotic chromosomes during all stages of mitosis (Figures 1 and 2c and Table 1). Thus all tested histone-modifying enzymes and chromatin-remodeling complexes, including ASH1, SU(VAR)3–9, BRM, OSA and ISWI, that are dissociated from DNA during replication are stably associated with DNA during mitosis.

### The number of protein–DNA foci decreases at late stages of mitosis

**Figure 3:**
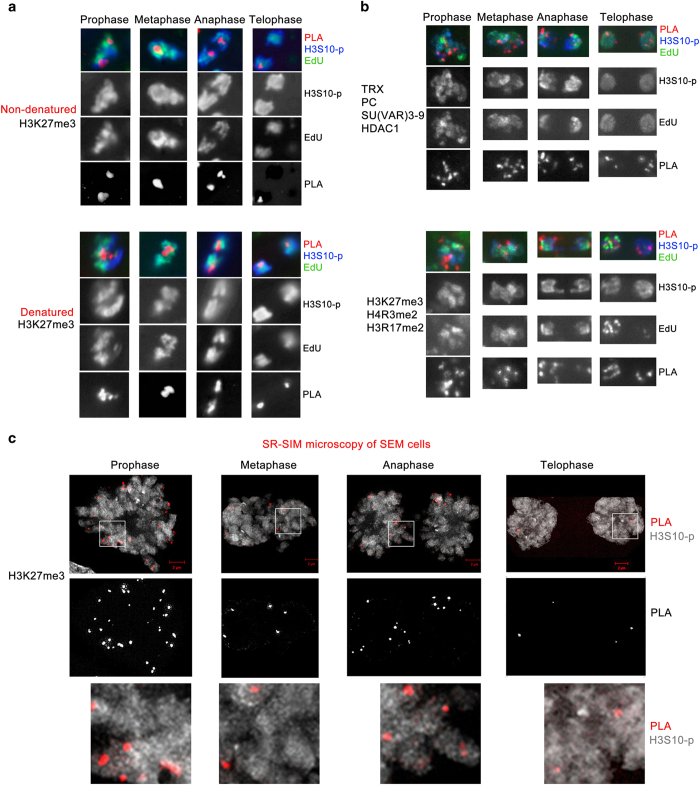
Number of protein-DNA foci decreases at late stages of mitosis. (**a**) Comparison of the number of proximity ligation assay (PLA) signals in Chromatin Association Assay (CAA) assays for H3K27me3 and 5-ethynyl-2′-deoxyuridine (EdU)-labeled DNA between a regular (top) and a denatured (bottom) sample. (**b**) CAA assays of mitotic nuclei with the indicated mixtures of chromosomal proteins (top) and modified histones (bottom). In (**a** and **b**), PLA, red; H3S10-p, blue; EdU, green. PLA signals only are shown in the bottom black and white panels. (**c**) Analysis of CAA in mammalian SEM cells using super-resolution microscopy (SR-SIM). Top panels, SR-SIM of the CAA assays for H3K27me3 in SEM cells. Scale bars are indicated. Middle panels, PLA signals only. Bottom panels, magnified SR-SIM images of nuclei shown in the top panels (white outline).

For most of the examined proteins, we detected a relatively low number of PLA signals on interphase and mitotic chromosomes; moreover, only a few signals are detected at later stages of mitosis. One explanation may be steric hindrance that leads to epitope masking, especially owing to condensation of chromatin in mitosis, thus reducing the number of PLA signals. We tested this by performing CAA experiments following heat denaturation of nuclei in formamide and found that this treatment did not increase the number of PLA signals for H3K27me3 at any stage of mitosis (Figure 3a). We concluded that a low number of DNA–protein signals cannot be explained by low accessibility of epitopes during mitosis. Another possibility is that there is a local jackpot effect for detecting epitope interactions on mitotic chromosomes. If this were the case, then all antibodies would detect the same, limited number of foci in our CAA assay. To examine this, we performed CAA experiments with a mixture of different antibodies to proteins known to be members of different protein complexes. As seen in Figure 3b, mixing antibodies against chromosomal proteins or modified histones in a single experiment leads to a significant increase in the number of PLA signals at each of the mitotic stages. Together, these results suggest that there are no technical limitations in detecting proteins through most of the regions of mitotic chromosomes by CAA.

The low number of PLA signals may also stem from the small sizes of nuclei of *Drosophila* embryos and the limit of resolution using conventional fluorescent microscopy. To improve the resolution of these experiments, we analyzed the association of H3K27me3 using super-resolution structured illumination microscopy (SR-SIM) [30] in mammalian SEM cells that have much larger nuclei than *Drosophila*. These assays yielded a significantly larger number of PLA signals in the prophase. However, despite much higher resolution of SR-SIM, we also detected a significant decrease in the number of PLA signals at later stages of mitosis (Figure 3c). We conclude that a low number of DNA–protein signals for a particular protein may reflect condensation of mitotic chromatin or some previously unrecognized features of mitotic chromosomes.

### Loss of H3K27 trimethylation does not affect the association of PRC1 or PRC2 with DNA

**Figure 4:**
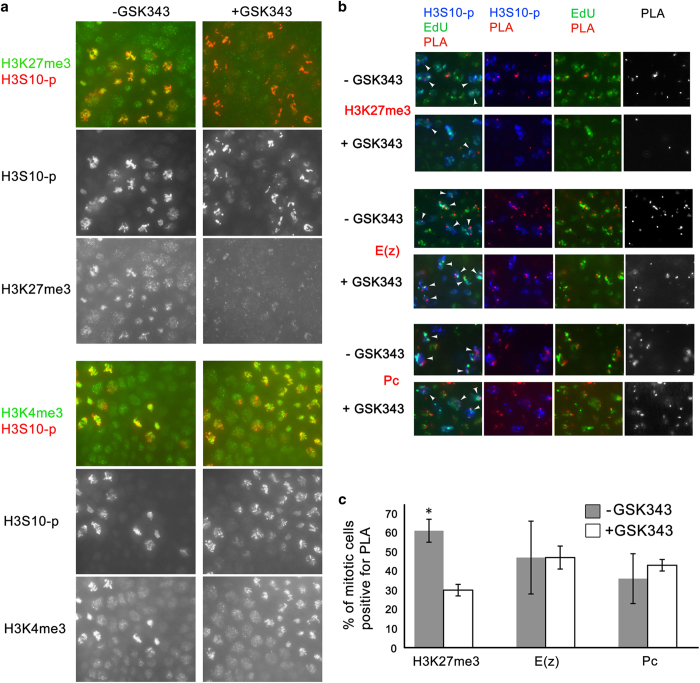
Reduced amount of H3K27me3 does not affect the association of E(Z) or PC with DNA. Embryos were untreated or treated with 50 μM of the E(z) inhibitor GSK343. (**a**) Embryos were immunostained with antibodies to H3K27me3 or H3K4me3 in green and H3S10-p in red. Split channels are indicated. (**b**) Chromatin Association Assay was performed between H3K27me3 (top), E(z) (middle) or Pc (bottom) and biotin (5-ethynyl-2′-deoxyuridine (EdU)). Proximity ligation assay (PLA), red; H3S10-p, blue; EdU, green. White arrowheads indicate PLA-containing nuclei that are labeled with EdU and H3S10-p. Pair-wise and single split channels are indicated. (**c**) Quantification of the results in (**b**). A statistically significant difference (*P*-value<0.05) is indicated by an asterisk (*).

It was proposed that H3K27me3 is essential for association of PcG PRC1 and PRC2 complexes following replication and mitosis [1, 31]. To determine whether the level of H3K27me3 affects the stable association of PRC2 and PRC1 subunits during mitosis, embryos were treated with the inhibitor of the only *Drosophila* H3K27me3 HMT E(Z), GSK343 [32, 33]. Treatment with GSK343 caused a significant decrease in the amount of nuclear H3K27me3 (Figure 4a). In control experiments, no difference in the amount of H3K4me3 was observed between treated and non-treated embryos, confirming the specificity of GSK343 for E(Z) (Figure 4a). We also detect a marked decrease in the number of PLA signals for DNA-associated H3K27me3 in both interphase and mitotic nuclei following GSK343 treatment (Figure 4b). The reduced H3K27me3 levels did not affect the number of PLA signals for PC and E(Z) (components of PRC1 and PRC2, respectively) in either mitotic or interphase nuclei (Figure 4b and c). Together, our results suggest that H3K27me3 is not essential for either recruitment or association of the PcG PRC1 and PRC2 complexes during mitosis.

### RNAs are associated with DNA during mitosis but are not essential for binding of PcG and TrxG proteins

**Figure 5:**
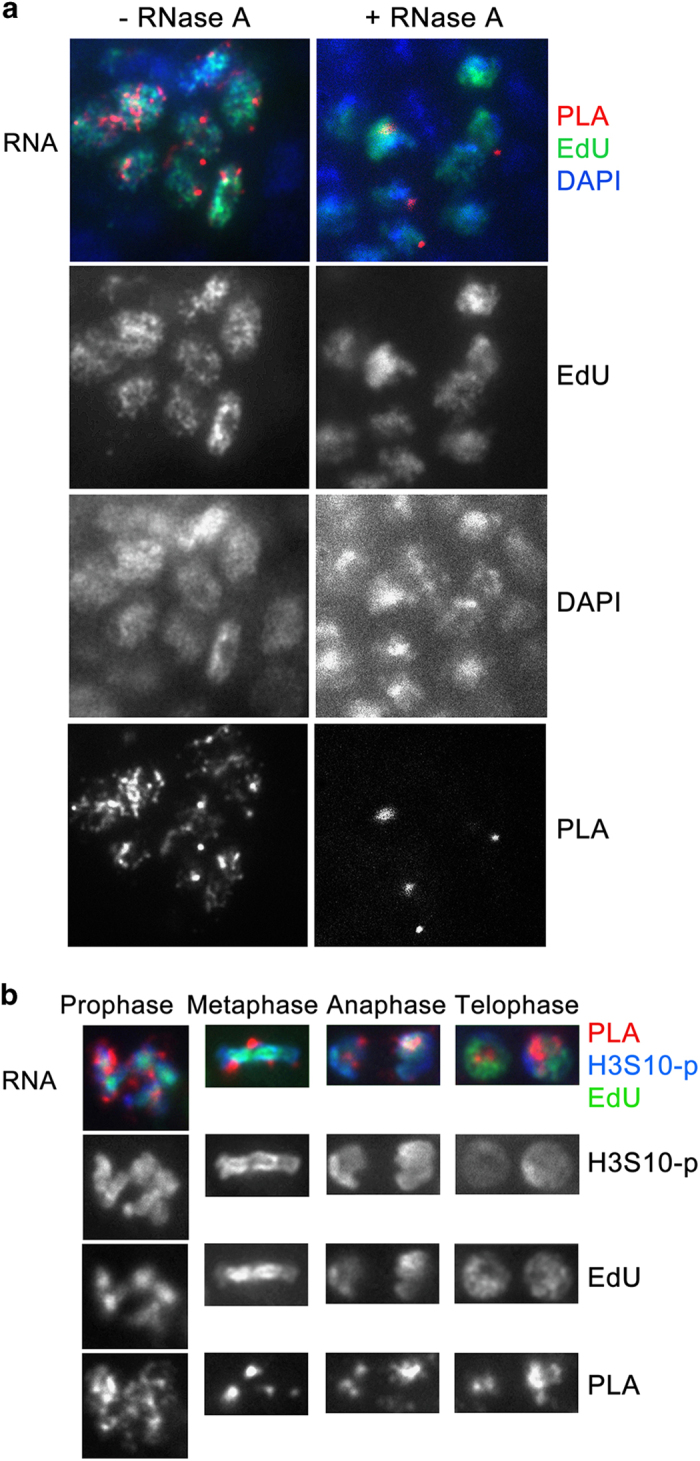
RNA is associated with DNA at all stages of mitosis. (**a**) Proximity of 5-bromouridine (BrU)-labeled RNA and 5-ethynyl-2′-deoxyuridine (EdU)-labeled DNA was assessed by RNA–DNA Interaction Assay (RDIA). Following labeling with BrU, embryos were either untreated (left) or treated (right) with RNase A. PLA, red; EdU, green; 4,6-diamidino-2-phenylindole (DAPI), blue. (**b**) Association of RNA with DNA at all mitotic stages was determined by RDIA. Black and white bottom panels show PLA signals only. PLA, red; EdU, green; H3S10-p, blue.

It was proposed that ncRNAs recruit PcG proteins to DNA following mitosis. However, the fate of any RNA during mitosis is unknown [34]. To assess this experimentally, we used the newly developed RNA–DNA Interaction Assay (RDIA) [16]. DNA in embryos was pulse-labeled with EdU for 30 min, chased for 1 h 40 min and then RNA was labeled with 5-bromouridine (BrU) for 15 min. RNA–DNA proximity was then detected by PLA (see below and Materials and Methods). The specificity of this assay was demonstrated previously [16] and is illustrated by the fact that multiple PLA signals between RNA and DNA are detected only in nuclei that are labeled by EdU (Figure 5a). Moreover, treatment of embryos with RNase almost completely eliminates PLA signals, further suggesting that these signals are specific to labeled RNA (Figure 5a). We found that in *Drosophila* embryos bulk RNAs are associated with DNA during all stages of mitosis (Figure 5b), similar to human lymphoblast cells (GM22737) examined previously [16].

**Figure 6:**
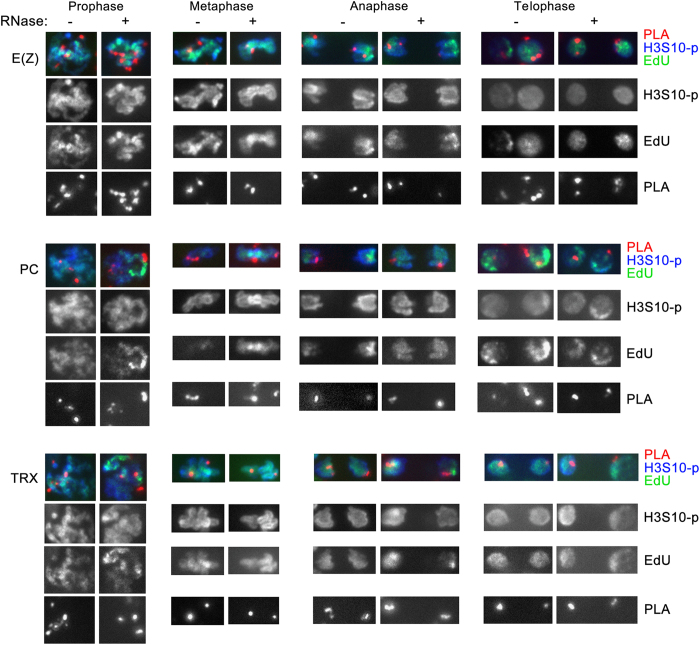
RNAs are not essential for association of E(Z), PC and TRX with mitotic chromosomes. Chromatin Association Assay was performed with antibodies to the indicated proteins and labeled DNA (biotin) in embryos that were either untreated (left columns) or treated with RNase A (right columns). Black and white bottom panels show proximity ligation assay (PLA) signals only. PLA, red; 5-ethynyl-2′-deoxyuridine (EdU), green; H3S10-p, blue.

RNAs are unlikely to be essential for recruitment of chromosomal proteins to DNA as proposed previously (discussed in Steffen and Ringrose [34]), as our CAA results suggest that these proteins are not dissociated from DNA through all mitotic stages. Nevertheless, the presence of RNA at mitotic chromosomes may imply that RNAs have a role in stabilizing chromosomal proteins at DNA during this stage of the cell cycle. To test this possibility, we assessed association of the PcG proteins PC and E(Z) and the TrxG protein TRX following extensive treatment of embryos with RNase. This treatment led to complete elimination of the PLA signals between RNA and DNA (Figure 5a), suggesting that most RNAs were destroyed. However, RNase treatment had no detectable effect on association of PC, E(Z) and TRX at any mitotic stage (Figure 6). Together, these results suggest that while RNAs are associated with DNA during mitosis and may thus have a certain epigenetic role, they are unlikely to be essential for either recruitment or association of chromosomal proteins during this stage of the cell cycle.

## Discussion

We used previously developed highly sensitive assays, RDIA [16] and CAA [28], to determine the association of DNA with RNA, methylated histones, chromatin-modifying enzymes and chromatin-remodeling proteins during mitosis. Compared with detection of proteins by conventional IF, these methods detect RNA or proteins within very close proximity (⩽40 nm) of DNA in intact nuclei at all stages of mitosis. RDIA is the only current method that tests the proximity of RNA and DNA *in vivo* at any stage of the cell cycle [16].

A common feature of our results is that the number of PLA signals is decreased 2–3-fold for all tested proteins during later stages of mitosis. This decrease is consistent with the recently determined degree of compaction of chromatin during mitosis [6, 35, 36]. Our experiments show that these changes are not caused by low resolution of conventional microscopy, the small size of nuclei in *Drosophila* embryos or reduced accessibility of epitopes in condensed chromatin. Instead, these results suggest that during mitosis individual chromatin proteins may be associated with nuclear compartments, similar to organization during interphase into chromatin domains, including transcribed (transcription factories) or repressed genes (PcG bodies) [6, 37, 38]. Our results also indicate that at later stages of mitosis these chromatin domains may undergo further reorganization, which is consistent with loss of long-range chromatin interactions [39].

A major finding of this work is that bulk RNAs, methylated histones and multiple chromatin factors, including PcG and TrxG epigenetic factors, representing both histone-modifying enzymes and chromatin-remodeling complexes, remain associated with DNA during all phases of mitosis (Figures 1 and 2, Table 1). We previously showed that methylated histones and some histone modifiers and nucleosome remodelers are dissociated from DNA during replication [28, 29]. However, their stable association with DNA during mitosis suggests a possible epigenetic role for these proteins and RNA during this stage of the cell cycle. These results are consistent with previous reports of transcriptional regulators that are partially or fully retained on mitotic chromatin (cited in Hsiung *et al.* [6]). Our findings strongly suggest that the stable DNA association of RNA, methylated histones and PcG- and TrxG-modifying and -remodeling enzymes during all stages of mitosis provides a mechanism for very complete conservation of most of the chromosomal proteins and RNA content in mitosis.

There is disagreement in the literature on PcG and TrxG protein association with mitotic chromosomes during *Drosophila* embryogenesis. One potential cause is developmental or cell-specific changes in the stability of these factors on chromosomes during mitosis. In preblastoderm embryos, EGFP (enhanced green fluorescent protein)-fusion proteins of PcG proteins E(Z), PHO and PC or endogenous Polyhomeotic [26, 40] were dissociated from chromosomes. However, there was detectable association with EGFP-fusion proteins of TrxG proteins TRX and ASH1 but not of OSA. In gastrulating embryos, Buchenau *et al.* [3] reported chromosome association of PcG proteins PSC and Polyhomeotic but not PC at metaphase followed by detection of all three proteins at anaphase. By contrast, our results on gastrulating embryos (Figures 1 and 2 and Table 1) show that all PcG and TrxG proteins remain stably associated with DNA at all stages of mitosis.

It is also possible that differences in the results stem from using differentiated S2 cells, which exit mitosis into the G1 phase, as opposed to cells of gastrulating embryos, which have no G1, and exit mitosis into the S phase. IF studies using *Drosophila* S2 cells derived from a primary culture of 20–24 h-old embryos [41] showed a difference in association between subunits of the PcG complexes, PRC1 and PRC2 [5]. The PRC1 subunits PSC, PC and dRING/SCE remain associated with chromosomes during all phases of mitosis while the PRC2 subunit E(Z) decreases during metaphase. In addition, similar results were obtained by ChIP-seq analysis of S2 cells arrested in mitosis [5]. We find no differences in DNA association between PRC1 and PRC2 subunits (Figure 1 and Table 1).

Other technical differences may also explain the discrepancies of our results with the literature, as the results of the previous IF studies suggest that HDAC1 dissociates from chromosomes at early mitotic stages and re-associates at late telophase [42, 43]. IF methods, even when advanced statistical analysis and confocal microscopy are used, can only show colocalization of antibody staining with 4,6-diamidino-2-phenylindole (DAPI) or mitotic markers, while CAA provides a direct assay of very close proximity, ⩽40 nm, of a protein and DNA. Furthermore, IF sensitivity is relatively low compared with the single-molecule sensitivity of CAA. The advantage of the ChIP assays is that they provide a direct test of the association of proteins with specific genomic loci. However, isolation of mitotic chromatin requires use of colcemid or nocodazole, which arrest cells in metaphase by disrupting microtubule assembly in condensed chromosomes [44]. Moreover, nocodazole-inhibited cells are not well synchronized [45], so ChIP analysis of protein binding is limited to prophase and metaphase and may potentially contain some G2 phase cells. Thus use of nocodazole may explain some differences in the modes of PRC1 and PRC2 subunit association with DNA during mitosis detected in this work and by others [5].

Similar to the previous reports [13, 25], we found that major modified histone forms are associated with DNA during all stages of mitosis. It has been proposed that histone modifications serve as platforms for protein recruitment or displacement during mitosis [31]. However, our results for H3K27me3 do not support this view. Subunits of PRC1 (PC) and PRC2 (E(Z)) complexes that recognize H3K27me3 are not displaced upon reduction of H3K27me3 levels by inhibition of the E(Z) HMT activity (Figure 4). Therefore, modified histone H3K27me3 is not essential for stable association of the PcG proteins PC and E(Z) on mitotic chromosomes. Moreover, we found that histone-modifying proteins and modified histones are associated with DNA during mitosis (Figures 1 and 2 and Table 1), suggesting that both types of proteins may have separate epigenetic role(s) during cell transition through mitosis. The role of modified histones is not related, however, to the stability or recruitment of histone-modifying proteins to postmitotic chromosomes as PC and E(Z) do not dissociate during mitosis.

We are not aware of any previous reports of the fate of RNAs during mitosis. Using a new method, RDIA, we found that in *Drosophila* embryos and in cultured mammalian cells [16] RNAs synthesized prior to mitosis remain in close proximity to DNA at all mitotic stages. Similar to proteins, compaction of chromatin during mitosis also reduces the number of RNA–DNA foci, suggesting major changes in the arrangement of specific chromatin domains during mitosis. The role of RNAs on mitotic chromosomes may be stabilization of proteins during chromosome condensation in mitosis. However, our results suggest that RNAs are not essential for association of PcG and TrxG proteins during mitosis (Figure 6). We conclude that proteins and RNAs are independently associated with DNA during mitotic stages. Together, our results suggest a possibility that association of transcripts and many essential chromosomal proteins on mitotic chromosomes may represent molecular bookmarking during mitosis required for inheritance of transcriptional states.

## Materials and Methods

### Analysis of protein–DNA association during mitotic stages in embryos (CAA)

Developmentally staged embryos were dechorionated in 50% bleach and permeabilized in octane. Nascent DNA was pulse labeled with 300 μM EdU in Schneider *Drosophila* Medium for 30 min followed by a 2 h chase (to label DNA in mitotic phase of the cell cycle). Embryos were washed and fixed in 1:1 mixture of 4% formaldehyde and heptane. EdU-labeled embryos were subjected to Click-iT reaction to attach biotin to EdU (using Click-iT Cell Reaction Buffer Kit, Life Science Tech, Carlsbad, CA, USA). Embryos were washed and incubated with goat monoclonal anti-biotin antibody (1:1 000, Vector, Burlingame, CA, USA) and the rabbit antibody of interest. PLA reaction was performed according to manufacturer’s instruction (Olink Bioscience, Uppsala, Sweden). Following incubation with primary antibodies from different species, secondary antibodies with conjugated oligonucleotides (MINUS and PLUS probes) were incubated for 1 h. Two oligonucleotides that hybridize to the two PLA probes were added, and DNA ligase joins them into a closed circle if they are in close proximity (up to 40 nm). PCR rolling circle amplification was carried out using phi29 polymerase in the presence of Alexa Fluor 594 fluorescent-labeled oligonucleotides (far red detector probe). Mitotic chromosomes were visualized by incubation with mouse monoclonal anti-H3S10-phosphorylated antibody (1:20 000, Abcam, Cambridge, MA, USA) followed by incubation with anti-mouse antibody conjugated with Alexa Fluor 555 (1:1 000, Life Technologies, Carlsbad, CA, USA). EdU-linked biotin was detected by incubation with Alexa Fluor 488-conjugated anti-goat antibody (1:1 000, Santa Cruz, Dallas, TX, USA).

### Analysis of RNA–DNA association during mitotic stages in embryos (RDIA)

After embryos were dechorionated and permeabilized in octane they were labeled with 200 μM EdU for 30 min followed by a chase for 1 h and 40 min in Drosophila Schneider medium. Embryos were then incubated with 2 mM BrU (Acros Organics, Geel, Belgium) in Drosophila Schneider medium for 15 min, washed in phosphate-buffered saline (PBS) and fixed. For RNase experiment, embryos were labeled with EdU, chased as described above and then incubated with 2 mM BrU (Acros Organics) in Drosophila Schneider medium for 5 min and Triton was added to final concentration 0.2% for additional 10 min of incubation. Embryos were briefly washed in PBT and treated for 20 min with either PBS (control treatment) or 1.5 mg ml^−1^ of RNase A (Roche, Basel, Switzerland) in PBS. Embryos were fixed and incubated first with Click-iT Cell reaction cocktail (Life Science Tech) for 30 min and then with goat monoclonal anti-biotin antibody (1:1 000, Vector) and mouse anti-bromodeoxyuridine antibody (1:200, BD Bioscience, San Diego, CA, USA) overnight at 4 °C. PLA assay was performed as described above. Mitotic chromosomes were visualized by incubation with rabbit anti-H3S10-phosphorylated antibody (1:800, Active Motif, Carlsbad, CA, USA) followed by incubation with anti-rabbit antibody conjugated with Alexa Fluor 555 (1:1000, Life Technologies). EdU-linked biotin was detected by incubation with Alexa Fluor 488-conjugated anti-goat antibody (1:1000, Santa Cruz).

### Treatment of embryos with the E(z) inhibitor GSK343

Embryos were pulse labeled with EdU for 30 min followed by chase for 2 h. During the entire labeling period, embryos were incubated in the medium containing 50 μM of the E(z) inhibitor GSK343 (Structural Genomics Consortium, Oxford, UK) dissolved in dimethyl sulfoxide. In control experiments, embryos were incubated for the same length of time in the presence of dimethyl sulfoxide without GSK343. Embryos were washed and fixed as described above.

### Heat denaturation of EdU-labeled embryos

Embryos were labeled with EdU for 30 min followed by chase for 2 h. EdU-labeled embryos were conjugated with biotin-azide using the Click-iT reaction as described earlier. Embryos were washed in PBS/0.3% Triton X-100 for 60 min at room temperature. They were subsequently incubated for 30 min each in PBS/0.3% Triton-X buffers containing 20, 50 and 80% of Prehybridization buffer (50% formamide/4× SSC/100 mM Na_2_HPO_4_, pH 7.0/0.1% Tween 20). After a final 30 min incubation in 100% Prehybridization buffer at room temprature, embryos were heated at 83 °C for 20 min in 100% Prehybridization buffer. Embryos were washed once in 50% formamide/2× SSC/0.3% CHAPS buffer (prewarmed at 37 °C) for 1 min, two times in 2× SSC/0.3% CHAPS buffer for 2 min and two times in PBS/0.3% Triton X-100 buffer for 2 min at room temperature. [46]. Embryos were incubated with antibodies and PLA probes as described earlier.

### Antibodies

The following antibodies were used (for unnamed sources, see Petruk *et al.* [29]: H3S10-p (1:20 000, Abcam); Trx (1:3 000); E(z) (1:2 000); Pc (1:2 000); H3K27me3 (1:4 000, Millipore, Darmstadt, Germany); H3K4me3 (1:15 000, Active Motif); H3K9me3 (1:2 000, Active Motif); H3R17me2 (1:2 000, Active Motif); H4R3me2 (1:4 000, Active Motif); PCNA (1:5 000, Abcam); ASH1 (1:1 000); dSET (1:500); LID (1:1 000); LSD (1:3 000); PHO (1:2 000); UTX (1:1 000), DART1 and DART4 (1:1 000 and 1:2 000, respectively); HDAC1 (1:2 000, Abcam); BRM (1:500); OSA and HP1 (1:3 000 and 1:5 000, respectively, Developmental Hybridoma Bank, Iowa City, IA, USA); ISWI (1:2 000, Abcam); Su(Var)3–9 (1:300, Abcam).

### Microscopy and analysis of embryos

Staged embryos were examined using fluorescence microscopy and SIM on a Zeiss ELYRA PS.1 (Carl Zeiss, Ithaca, NY, USA). SIM *z*-stacks were acquired with five rotations and five phase shifts with a ×63 Plan-Apochromat (NA=1.4). SR-SIM reconstructions were performed using a theoretical point spread function and an affine image alignment based on a multi-speck bead alignment. Comparative wide-field images were generated from the SIM *z*-stacks before processing. Specificity of PLA was monitored by examining whether PLA signals are detected only in the EdU-labeled nuclei. Pictures were taken of several embryos from at least three independent experiments and pictures used are a representative selection. To calculate the percentages for mitotic and non-mitotic nuclei, 100 mitotic and non-mitotic nuclei from 5 different embryos for each antibody were used. A nucleus containing at least one PLA signal was counted as a positive. A two-tailed independent *t*-test was performed for each antibody’s mitotic and non-mitotic data to generate a *P*-value. *P*-values <0.05 were considered to be significant.

## Figures and Tables

**Table 1:** Number of PLA signals for chromosomal proteins and modified histones on labeled DNA during interphase and mitotic stages

	*Interphase*	*Prophase*	*Metaphase*	*Anaphase*	*Telophase*
H3S10p	ND	6.1±2.7	4.8±2.4	6.3±1.8	1.8±1.2
TRX	3.1±1.9	1.8±0.8	2.2±1.3	1.6±0.9	1.6±0.7
E(Z)	2.6±1.9	2.1±1.1	1.5±1.2	1.2±0.4	1.1±0.3
PC	2.8±1.3	1.8±0.8	2.0±1.0	1.4±0.7	1.3±0.5
ASH1	4.3±2.0	2.2±0.9	1.5±1.0	1.9±0.8	1.3±0.4
SETD1	4.3±1.7	3.9±1.1	2.4±0.9	4.3±2.1	1.6±0.6
PHO	2.2±1.2	2.3±1.0	1.8±0.8	1.1±0.4	1.4±0.7
SU(VAR)3–9	4.0±1.8	3.3±1.9	1.8±1.0	2.5±2.0	1.4±0.6
DART1	1.9±1.0	1.3±0.5	1.1±0.4	1.3±0.6	1.1±0.3
DART4	2.6±1.3	1.9±1.2	1.5±0.6	1.4±0.5	1.3±0.5
LID	1.6±0.7	1.1±0.4	1.2±0.4	1.3±0.6	1.2±0.4
LSD1	1.8±0.7	1.6±0.8	1.3±0.6	1.0±0.0	1.0±0.0
UTX	2.0±0.9	2.0±1.1	1.3±0.6	1.3±0.5	1.2±0.4
HDAC1	3.0±1.4	1.7±1.2	1.3±0.5	1.5±0.5	1.1±0.4
CBP	2.3±1.2	1.6±0.8	1.4±0.5	1.2±0.4	1.6±0.7
pCAF	2.8±1.3	2.1±1.1	1.6±0.5	1.7±1.3	1.4±0.5
ISWI	4.2±2.2	4.2±1.6	3.3±1.7	2.0±1.2	1.3±0.8
BRM	2.1±1.0	1.4±0.5	1.6±0.5	1.4±0.5	1.5±0.8
H3K27me3	4.0±2.1	2.2±1.5	1.2±0.5	1.9±1.0	1.3±0.5
H3K4me3	2.3±1.2	2.5±1.7	1.7±0.9	1.9±1.0	1.3±0.6
H3K9me9	1.9±0.7	1.4±0.6	1.2±0.4	1.1±0.3	1.1±0.5
H3R17me2	2.4±1.1	1.8±1.0	1.3±0.5	1.2±0.4	1.7±0.7
H4R3me2	2.5±1.4	1.9±1.1	1.3±0.6	1.2±0.4	1.1±0.4

Abbreviations: ND, not determined because of the low number of PLA-positive nuclei; PLA, proximity ligation assay. The number of PLA signals (mean±s.d.) for each of the above proteins during interphase and each phase of mitosis were quantified in 50–200 nuclei from three independent experiments.
